# Dual Nature of Mitochondrial Integrated Stress Response: Molecular Switches from Protection to Pathology

**DOI:** 10.3390/genes16080957

**Published:** 2025-08-13

**Authors:** Jisu Jeong, Junghyun Kim, Man S. Kim

**Affiliations:** 1 Translational-Transdisciplinary Research Center, Medical Science Research Institute, Kyung Hee University Hospital at Gangdong, College of Medicine, Kyung Hee University, Seoul 02447, Republic of Korea; symply501@khu.ac.kr; 2 Department of Biomedical Science and Technology, Graduate School, Kyung Hee University, Seoul 02447, Republic of Korea; 3 Division of Tourism & Wellness, Hankuk University of Foreign Studies, Yongin-si 17035, Gyeonggi-do, Republic of Korea

**Keywords:** integrated stress response, mitochondrial dysfunction, eIF2α phosphorylation, cellular adaptation, neurodegeneration, precision medicine

## Abstract

Background: The mitochondrial integrated stress response (ISR) represents a fundamental cellular adaptation mechanism with dual protective and pathological roles. We critically analyzed current literature on ISR mechanisms, focusing on recent paradigm shifts including the 2020 discovery of the OMA1-DELE1-HRI axis, emerging controversies over context-dependent activation patterns, and the January 2025 clinical trial failures that have reshaped the therapeutic landscape. Methods: We reviewed recent literature (2020–2025) examining ISR mechanisms, clinical trials, and therapeutic developments through comprehensive database searches. Results: The field has evolved from simple linear pathway models to recognition of complex, context-dependent networks. Recent findings reveal that ISR activation mechanisms vary dramatically based on cellular metabolic state, with distinct pathways operating in proliferating versus differentiated cells. The “dark microglia” phenotype in neurodegeneration and DR5-mediated apoptotic switches exemplify pathological ISR manifestations, while adaptive responses include metabolic reprogramming and quality control enhancement. Conclusions: The 2025 failures of DNL343 and ABBV-CLS-7262 in ALS trials underscore the need for precision medicine approaches that account for context-dependent ISR functions, temporal dynamics, and disease-specific mechanisms.

## 1. Introduction

The integrated stress response (ISR) represents one of the most sophisticated cellular adaptation networks in biology, coordinating responses to diverse stresses through conserved eIF2α phosphorylation mechanisms [1]. The historical development of ISR understanding began with independent discoveries of individual eIF2α kinases: GCN2 in yeast amino acid starvation responses (1992) [2], PERK in endoplasmic reticulum stress (1999) [3], PKR in antiviral immunity [4], and HRI in heme deficiency [5]. The “integrated” concept emerged from recognition that these disparate pathways converge on a shared translational control mechanism [6,7].

The 2020 breakthrough discovery of the OMA1-DELE1-HRI axis by Guo et al. and Fessler et al. [8,9] fundamentally transformed our understanding of mitochondrial-cytoplasmic communication. This mechanism provided the long-sought “missing link” connecting mitochondrial dysfunction to ISR activation, revealing how mitochondrial membrane depolarization triggers cytoplasmic stress responses through DELE1 cleavage and release.

However, recent critical analyses have challenged the universality of this model. Mick et al. demonstrated that ISR activation is profoundly context-dependent rather than universal [10]. In proliferating cells, complex I inhibition activates ISR through asparagine depletion and GCN2, completely bypassing the OMA1-DELE1-HRI pathway. In differentiated cells, different mechanisms predominate, revealing that cellular metabolic state fundamentally determines which ISR pathway is activated [10].

This complexity has profound clinical implications, highlighted by the January 2025 failures of two major ISR-targeted therapeutics in Phase 2/3 ALS trials [11,12]. DNL343 (Denali Therapeutics) and ABBV-CLS-7262/Fosigotifator (Calico/AbbVie) both failed to meet primary endpoints despite strong preclinical evidence and good target engagement, marking a critical inflection point for the field [11,12].

The dual nature of ISR emerges from its role as both cellular protector and potential pathological driver [13]. Under moderate stress conditions, ISR promotes survival through metabolic reprogramming, enhanced quality control, and adaptive transcriptional responses [14]. However, when activation exceeds critical thresholds—particularly with chronic stimulation or excessive eIF2α phosphorylation—the same pathways become instruments of cellular demise through DR5-mediated apoptosis [15,16]. Understanding these molecular switches between protection and pathology is crucial for developing effective ISR-targeted therapeutics.

## 2. Molecular Architecture of Mitochondrial ISR Signaling

### 2.1. OMA1-DELE1-HRI Pathway Discovery and Mechanism

The identification of DELE1 (DAP3-binding cell death enhancer 1) as a critical mediator between mitochondrial dysfunction and cytoplasmic ISR activation represented a major breakthrough [8,9]. Genome-wide CRISPR screens revealed DELE1 as the missing link connecting mitochondrial membrane potential loss to HRI activation and subsequent eIF2α phosphorylation [8].

DELE1 exists as a dual-localized protein with full-length DELE1 (DELE1L) associated with the inner mitochondrial membrane and processed short forms (DELE1S) accumulating in the cytosol during stress [17]. The canonical mechanism involves OMA1 protease activation following mitochondrial depolarization, cleaving DELE1 during import or from membrane pools [8,9]. However, recent structural studies revealed additional complexity: DELE1 C-terminal domains assemble into octameric oligomers with D4 symmetry, creating sophisticated signaling platforms for HRI activation [18]. Oligomerization-deficient mutants can bind HRI but cannot activate ISR, adding crucial regulatory layers [18].

Critically, multiple proteases beyond OMA1 can generate functional DELE1 fragments, with cell-type-specific involvement suggesting far more complex regulation than originally proposed [19]. Furthermore, iron-responsive DELE1 pathways operating independently of OMA1 cleavage have been discovered, revealing “a hitherto unappreciated mitochondrial-based iron-monitoring system” [20].

### 2.2. Context-Dependent Activation: Metabolic State Paradigm

A paradigm shift has emerged from recognition that ISR activation mechanisms vary dramatically based on cellular context [10]. In proliferating myoblasts, complex I inhibition (rotenone treatment) activates ISR through asparagine depletion and GCN2 kinase activation, completely bypassing the OMA1-DELE1-HRI pathway. In contrast, differentiated myotubes respond to ATP synthase inhibition through mitochondrial membrane hyperpolarization via entirely different mechanisms [10].

This context dependency extends to other cellular states. Nutrient-rich versus nutrient-depleted conditions activate distinct ISR arms, with implications for therapeutic targeting [21]. The discovery challenges decades of simplified linear pathway models and suggests that cellular metabolic state fundamentally determines ISR pathway selection [10].

Plant systems provide evolutionary perspective on this complexity. Unlike mammals with four eIF2α kinases, plants rely solely on GCN2 [22]. Remarkably, eIF2α phosphorylation in plants does not significantly inhibit global protein synthesis, contrasting sharply with the mammalian paradigm [23]. This evolutionary divergence suggests the four-kinase mammalian system represents specialized adaptation for complex multicellular stress responses.

### 2.3. Technical Challenges and Research Limitations

Recent revelations have exposed fundamental flaws in ISR research methodology. Szaruga et al. demonstrated that ATP-competitive inhibitors of one ISR kinase paradoxically activate sister kinases at micromolar concentrations commonly used in studies [24]. PERK inhibitors GSK2656157, GSK2606414, and AMG44 activate GCN2, while GCN2 inhibitor A92 activates PERK through increased ATP affinity mechanisms [24]. This cross-activation potentially invalidates conclusions from numerous preclinical studies using these inhibitors at cross-activating concentrations.

Quantitative ISR analysis reveals critical activation thresholds. Physiological eIF2α phosphorylation increases of 2–3 fold support cell survival, whereas sustained increases exceeding 5-fold consistently trigger cell death pathways [25]. CHOP expression serves as a critical rheostat: basal levels (<2-fold increase) are protective, intermediate levels (2–10 fold) yield context-dependent outcomes, and sustained high expression (>10-fold) reliably drives apoptosis [26].

## 3. Protective ISR Mechanisms: Adaptive Cellular Responses

### 3.1. Metabolic Reprogramming and Amino Acid Homeostasis

ISR orchestrates comprehensive metabolic reprogramming supporting cellular survival during mitochondrial dysfunction [14]. ATF4-mediated transcriptional programs fundamentally rewire amino acid metabolism, particularly enhancing serine biosynthesis through upregulation of PHGDH, PSAT1, and PSPH enzymes [27]. This metabolic shift redirects carbon flux from glycolysis toward serine production, supporting one-carbon metabolism crucial for nucleotide synthesis and stress responses [14].

The transsulfuration pathway receives particular emphasis during ISR activation, with CTH enzyme upregulation increasing cysteine availability for glutathione synthesis [28]. Studies demonstrate that ISR activation diverts 3-phosphoglycerate from energy production toward amino acid synthesis, markedly increasing serine biosynthesis while reducing tricarboxylic acid cycle flux and fatty acid synthesis [29]. This metabolic rewiring provides essential building blocks for stress response proteins while conserving energy for survival.

Lipid metabolism reorganization through DGAT-dependent lipid droplet formation represents another protective mechanism [30]. DGAT2-mediated triglyceride synthesis and lipid droplet accumulation serve protective functions by buffering cellular stress and maintaining membrane integrity against oxidative damage [30].

### 3.2. Quality Control Integration and Proteostasis Enhancement

ISR integrates seamlessly with mitochondrial quality control mechanisms, particularly the mitochondrial unfolded protein response (UPRmt) and selective autophagy pathways [31]. The UPRmt operates as an integrated ISR component, with both pathways sharing transcriptional regulators including ATF4, ATF5, and CHOP to ensure coordinated proteostasis management [32].

A unique discovery reveals that HRI-mediated ISR specifically induces mitophagy through phosphorylated eIF2α mitochondrial localization, sufficient to trigger selective mitochondrial autophagy [33]. This HRI-mitophagy pathway operates independently of the classical PINK1/Parkin pathway, providing alternative mechanisms for damaged mitochondrial removal [33].

CHOP serves as a critical negative feedback regulator, preventing ISR overactivation through interaction with C/EBPβ to regulate ATF4 levels [34]. This sophisticated regulatory network ensures that quality control responses remain proportional to stress levels, preventing excessive activation that could compromise cellular function [35].

### 3.3. Antioxidant Defense and Cellular Protection Mechanisms

ISR activation provides robust protection against oxidative damage through multiple coordinated mechanisms [36]. Enhanced cysteine availability through the ATF4-CTH axis supports glutathione synthesis, while hydrogen sulfide production provides persulfidation-mediated protection against oxidative stress [28]. This dual strategy involves both direct reactive oxygen species scavenging and protein protection through post-translational modifications.

Global translation inhibition conserves energy while selectively preserving synthesis of stress-responsive proteins [3]. This selective translation program, mediated by upstream open reading frames, ensures continued production of essential stress response factors including ATF4, ATF5, and antioxidant enzymes while reducing overall metabolic burden [37].

## 4. Pathological ISR Mechanisms and Cellular Toxicity

### 4.1. DR5-Mediated Cell Death and the ISR Kill Switch

Recent studies have identified death receptor 5 (DR5) as a central mediator of ISR-induced cell death, representing a critical molecular switch that eliminates irreversibly injured cells [15]. Lu et al. first demonstrated that opposing unfolded protein response signals converge on DR5 to regulate apoptosis, revealing a novel paradigm where DR5 can be activated solely through increased expression during ISR, independent of extracellular ligands [15].

This pathway involves persistent PERK activation during unrelenting stress, leading to CHOP expression that directly upregulates DR5 transcription [16]. Complete abolition of ISR-induced cell death occurs when DR5 is genetically depleted, confirming its essential role [38]. DR5 upregulation occurs across multiple ISR-activating stresses, leading to its designation as a “general ISR kill switch” [39].

**Figure 1:**
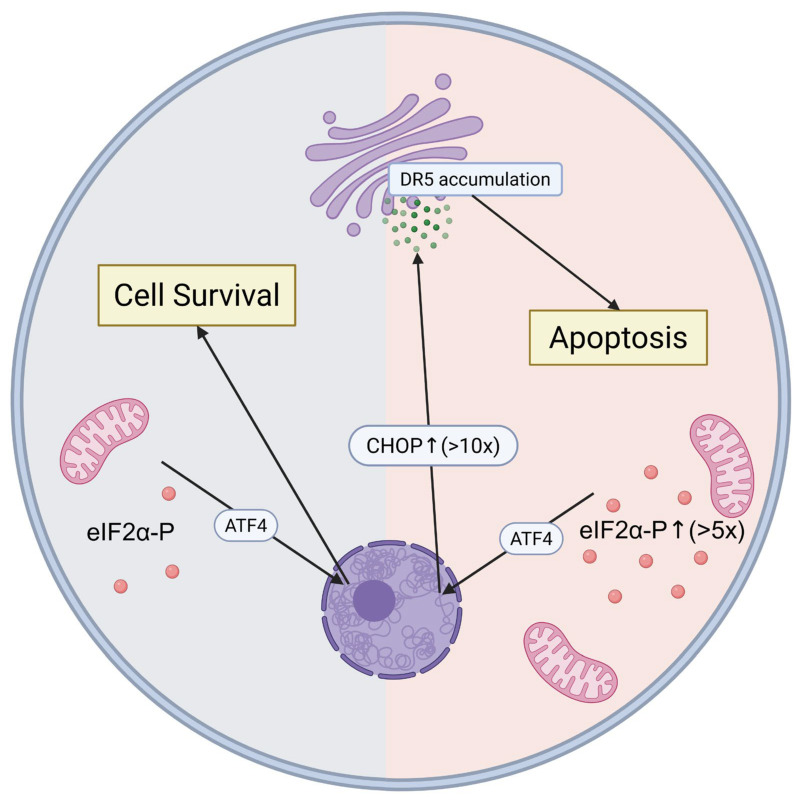
DR5-Mediated Apoptotic Switch in Pathological ISR Activation. The integrated stress response (ISR) operates as a molecular switch determining cell fate based on eIF2α phosphorylation levels. Under mild stress (left), low eIF2α-P activates ATF4 for adaptive cell survival responses. When eIF2α phosphorylation exceeds critical thresholds (>5-fold, right), sustained ATF4 activation leads to massive CHOP upregulation (>10-fold). CHOP directly upregulates DR5 expression, leading to DR5 accumulation in the Golgi apparatus and ligand-independent activation of the extrinsic apoptotic pathway. This quantitative threshold mechanism explains how the same ISR pathway promotes either cellular protection or programmed cell death, representing the ISR’s dual nature as both cellular guardian and executioner. Created in BioRender. (2025) https://BioRender.com/f6won2a (accessed on 31 July 2025).

Recent mechanistic studies by Zappa et al. revealed that this represents a cell-autonomous apoptotic program where DR5 oligomerization occurs independent of ligand binding, triggered solely by ISR-mediated expression increases [40]. This binary switch mechanism activates once ISR surpasses critical thresholds, marking a point of no return where adaptive responses transition to programmed cell death [15] (Figure 1).

### 4.2. Inflammatory Cascades and Tissue Damage Mechanisms

Pathological ISR activation triggers systemic inflammatory responses extending cellular damage beyond initially stressed cells [41]. Excessive ISR contributes to systemic inflammatory response syndrome through cytokine production, leading to tissue damage, hemodynamic changes, and organ failure [42]. Mitochondrial-derived damage-associated molecular patterns, including mtDNA and formyl peptides, serve as alarmins triggering systemic inflammation when released from ISR-compromised cells [43].

Neuroinflammation represents a particularly significant consequence where ISR-activated microglia produce and secrete toxic lipids damaging neurons and oligodendrocyte progenitor cells [44]. This process results in blood-brain barrier breakdown and peripheral immune cell recruitment, amplifying neuroinflammatory responses beyond initial mitochondrial stress [44]. The inflammatory cascade involves hyperactivation of NF-κB, MAPK, and JAK-STAT pathways, with chronic eIF2α phosphorylation disrupting protein homeostasis and promoting misfolded protein accumulation that serves as additional inflammatory triggers [45].

### 4.3. Dark Microglia and Neurodegeneration Mechanisms

The discovery of “dark microglia” in Alzheimer’s disease represents a striking example of ISR pathology in neurodegeneration [44]. Dark microglia are defined as ultrastructurally distinct microglial cells characterized by electron-dense cytoplasm and nuclear condensation, detectable only through electron microscopy [46]. These cells are present at twice the levels in Alzheimer’s disease patients compared to healthy aged individuals [47].

Autonomous ISR activation in microglia is sufficient to induce early dark microglial features, establishing a direct mechanistic link between cellular stress responses and neuroinflammatory pathology [44]. ISR-activated microglia produce and release toxic lipids, including ceramides and sphingolipids, contributing to synapse loss characteristic of Alzheimer’s disease [44]. This represents a non-cell-autonomous stress mechanism where ISR activation in microglia drives pathology in neurons, demonstrating the systemic nature of ISR-mediated damage.

Dark microglia accumulate around amyloid plaques and neurofibrillary tangles, suggesting involvement in disease-specific pathological processes [47]. Pharmacological studies reveal therapeutic potential through ISR or lipid synthesis pathway inhibition, which mitigates synaptic loss in Alzheimer’s models [44]. The reversibility of symptoms upon ISR inhibition in preclinical models offers promise while underscoring the critical importance of therapeutic timing [48].

## 5. Disease Contexts and Clinical Implications

### 5.1. Neurodegeneration and the ISR Paradox

Neurodegenerative diseases exhibit distinct ISR activation patterns and temporal dynamics [49]. Alzheimer’s disease involves dual PERK/PKR activation with tau-centric pathology, primarily affecting hippocampal neurons and astrocytes [50]. Post-mortem brains show significantly increased eIF2α phosphorylation and reduced eIF2B levels in cortical and hippocampal regions [50]. Initially adaptive responses to amyloid-β-induced stress become pathological through chronic activation, leading to persistent translation suppression and BACE1 upregulation, creating positive feedback loops increasing amyloid-β production [51].

Parkinson’s disease demonstrates PERK-ATF4 dominance with mitochondrial focus and HRI-dependent mitophagy activation [52]. The PERK-ATF4 pathway initially promotes survival through Parkin transcription, representing protective responses to mitochondrial stress and oxidative damage [53]. However, excessive ATF4 activity promotes pro-apoptotic factors including CHOP and Trib3, contributing to dopaminergic neuronal death [54].

Amyotrophic lateral sclerosis (ALS) presents predominantly PERK-mediated ISR activation with motor neuron-specific vulnerability patterns [55]. Motor neurons show elevated p-eIF2α, ATF4, CHOP, and BiP, with protein aggregation involving TDP-43, SOD1, and FUS triggering ISR activation [56]. Fast-fatigable motor neurons demonstrate greater vulnerability than slow motor units, suggesting cell-type-specific susceptibility [57].

A unifying theme across neurodegenerative diseases involves ISR transition from protective to pathological over time [58]. This temporal switch likely explains conflicting research findings and highlights why therapeutic timing is crucial for clinical success.

**Figure 2:**
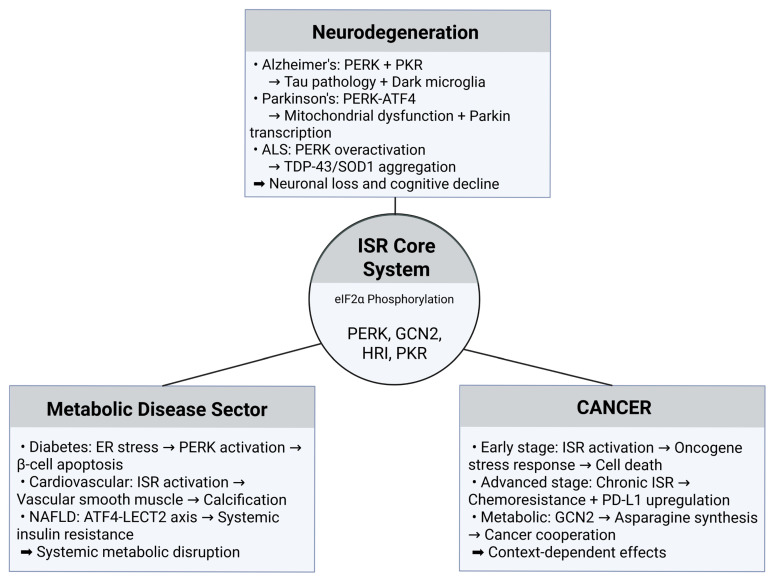
Context-dependent ISR signaling across disease categories. ISR operates through common eIF2α phosphorylation but produces distinct outcomes across diseases. In neurodegeneration, ISR contributes to protein pathology (tau, TDP-43/SOD1 aggregation) and cellular dysfunction (dark microglia, mitochondrial damage). In cancer, ISR shows dual roles as tumor suppressor (early) or promoter (advanced), with metabolic reprogramming enabling cancer cell cooperation. In metabolic disorders, ISR causes organ-specific dysfunction through β-cell apoptosis, vascular calcification, and systemic insulin resistance. This context-dependency necessitates disease-specific therapeutic approaches. Created in BioRender. (2025) https://BioRender.com/f6won2a (accessed on 31 July 2025).

The context-dependent nature of ISR activation across different disease categories is illustrated in Figure 2. Despite sharing common eIF2α phosphorylation mechanisms, ISR produces dramatically different pathological outcomes depending on cellular context, disease progression, and tissue-specific factors. This mechanistic complexity provides crucial insights into why disease-agnostic ISR therapeutic approaches have faced significant challenges in clinical translation, emphasizing the need for precision medicine strategies that account for disease-specific ISR manifestations.

### 5.2. Cancer Biology and Metabolic Reprogramming

Cancer biology reveals complex ISR roles in tumor progression, serving both tumor-suppressive and promotional functions depending on cellular context and oncogenic drivers [58]. In KRAS-driven lung adenocarcinoma, ISR promotes tumor growth and survival by enabling adaptation to hostile tumor microenvironments characterized by nutrient deprivation, hypoxia, and metabolic stress [59]. ISR facilitates metabolic reprogramming necessary for cancer cell survival while promoting therapeutic resistance and immune escape through PD-L1 upregulation [60].

Conversely, ISR can function as a tumor suppressor when activation levels exceed cellular tolerance [61]. Excessive ISR activation overwhelms adaptive mechanisms, promotes apoptosis through CHOP upregulation, and sensitizes cancer cells to therapeutic interventions [62]. This dual role creates opportunities for context-dependent therapeutic strategies where ISR inhibition may benefit some cancer types while ISR activation could be advantageous in others [63].

Cancer stem cells preferentially utilize ISR for survival under stress conditions, with higher baseline ISR activity correlating with therapeutic resistance [64]. This finding suggests that ISR-targeted approaches may be particularly effective against treatment-resistant cancer populations.

### 5.3. Cardiovascular and Metabolic Disorders

Cardiovascular disease involves ISR through multiple mechanisms, with hypoxic conditions in failing hearts triggering PERK activation while metabolic stress activates GCN2 pathways [65]. ISR contributes to vascular calcification through PERK/eIF2α/ATF4 signaling leading to vascular smooth muscle cell osteoblastic differentiation, resulting in arterial stiffening and increased cardiovascular mortality [66].

Type 2 diabetes exemplifies dual ISR roles in metabolic disorders [67]. Initially, ISR serves as an adaptive response to metabolic stress in pancreatic β-cells, maintaining glucose homeostasis and supporting insulin secretion under mild stress conditions [68]. However, chronic ISR activation contributes to β-cell dysfunction and death while impairing insulin sensitivity in peripheral tissues and promoting diabetic complications [69].

Metabolic syndrome, affecting approximately 25% of the global population, shows strong associations with ISR activation through connections between insulin resistance and cellular stress responses [70]. ER stress in adipose tissue triggers PERK activation while inflammatory cytokines activate PKR pathways, creating feed-forward cycles where metabolic dysfunction promotes additional ISR activation [71].

## 6. Clinical Therapeutic Development and Evolving Strategies

### 6.1. The 2025 Clinical Trial Catastrophe

January 2025 marked a devastating setback for ISR therapeutics with simultaneous Phase 2/3 failures in the HEALEY ALS Platform Trial [11,12]. These failures have fundamentally reshaped the field’s understanding of ISR as a therapeutic target.

DNL343 (Denali Therapeutics) was evaluated in 186 treated versus 139 placebo patients [11]. The eIF2B activator showed complete failure with no differences in disease progression, survival, or biomarker endpoints. The extension study was discontinued early, and the company is “mulling the asset’s future” [11]. Despite robust preclinical evidence and confirmed target engagement, the compound failed to demonstrate any clinical benefit.

ABBV-CLS-7262/Fosigotifator (Calico/AbbVie) involved 234 treated versus 126 placebo patients [12]. The primary endpoint failed with no effect on disease progression. However, exploratory high-dose analysis showed potential muscle strength benefits (32% upper extremity improvement in a subset), leading to continued development in other indications [12].

These failures occurred despite strong preclinical data, good safety profiles, and confirmed pathway modulation, raising fundamental questions about ISR as a therapeutic target [72]. The disconnect between target engagement and clinical efficacy suggests that ISR modulation alone may be insufficient to address disease complexity in ALS [73] (Table 1).

### 6.2. ISRIB: Promise and Limitations

ISRIB (Integrated Stress Response Inhibitor) functions through allosteric stabilization of the eIF2B complex to overcome inhibitory effects of phosphorylated eIF2α [74]. Originally discovered through phenotypic screening, ISRIB binds to the eIF2B regulatory core, promoting conformational changes that reduce sensitivity to p-eIF2α inhibition [75].

Clinical applications show promise in multiple therapeutic areas [76]. Memory enhancement studies demonstrate that ISRIB improves cognitive performance in healthy animals and reverses memory deficits in models of traumatic brain injury, aging, and neurodegeneration [48]. The compound effectively crosses the blood-brain barrier with favorable safety profiles in preclinical studies [77].

However, ISRIB’s therapeutic window appears narrow, with efficacy limited to conditions where eIF2α phosphorylation levels remain below critical thresholds [78]. Studies demonstrate that ISRIB loses effectiveness when p-eIF2α levels exceed 45–70% of maximum phosphorylation, suggesting molecular switches determining drug responsiveness [79]. Clinical development has been remarkably slow since 2021, with reports that “its efficacy vs. safety profile may be compromised by side effects” [80].

### 6.3. Alternative Therapeutic Strategies and Future Directions

Despite setbacks in neurodegeneration, ISR-targeted therapies show promise in other contexts [82]. ISRIB combined with imatinib enhanced treatment sensitivity and eliminated TKI-resistant chronic myeloid leukemia blast cells by specifically inhibiting STAT5 and RAS/RAF/MEK/ERK pathways [83]. This combination demonstrated superior efficacy compared to monotherapy, providing templates for successful ISR-targeted approaches.

Combination strategies represent the most promising future direction. ISRIB combined with gemcitabine overcame chemoresistance in pancreatic cancer by blocking cytoprotective ISR activation and inducing enhanced apoptosis compared to gemcitabine alone [84]. This highlights potential for ISR inhibition to enhance conventional therapies by preventing adaptive stress responses promoting drug resistance [85].

Cell-type-specific targeting approaches are emerging to address ISR’s context-dependent functions. Strategies using tissue-specific delivery systems or cell-type-selective compounds may overcome limitations of systemic ISR modulation [86]. Temporal modulation based on disease stage represents another promising avenue, with early intervention potentially preserving protective ISR functions while later intervention might target pathological aspects [87].

## 7. Precision Medicine: Biomarkers and Patient Stratification

### 7.1. Current Biomarker Landscape and Clinical Applications

The precision medicine toolkit for ISR therapeutics centers on several validated biomarkers [88]. Primary markers include p-eIF2α (measurable via commercial AlphaLISA platforms), ATF4 (nuclear translocation indicates ISR activation within 2 h), and CHOP (pro-apoptotic marker for pathological ISR) [89]. Emerging markers encompass GDF-15 and FGF-21 (mitokines indicating mitochondrial stress), N-lactoyl-amino acids (novel biomarkers in mitochondrial diseases), and imaging approaches using 18F-BCPP-EF PET for respiratory chain activity [90].

Disease-specific stratification strategies show promise but face implementation challenges [91]. In cancer, high p-eIF2α in tumors correlates with better prognosis and enhanced antitumor immunity, while PTEN loss/MYC amplification in prostate cancer predicts ISRIB response [92]. For neurological disorders, baseline ISR activation levels in CSF or brain tissue may predict therapeutic response, though clinical validation remains limited [7].

Critical gap: Despite available technologies, no FDA-approved ISR companion diagnostics exist, reflecting translational challenges facing the field [8]. The development of validated, non-invasive biomarkers for patient ISR status remains a critical barrier to clinical translation.

### 7.2. Patient Selection and Stratification Strategies

Genetic stratification approaches selecting patients with mutations causing pathway hyperactivation have shown enhanced response rates [81]. Specific patient subsets demonstrate particular benefits from combination therapies targeting hyperactivated signaling cascades [81]. ATF4 expression levels are being developed as predictive biomarkers for therapeutic responses, enabling precision medicine approaches [93].

AI and machine learning integration of multi-omics data shows promise for patient stratification, with Alzheimer’s trials demonstrating 46% improvement in outcomes for precisely stratified patients [94]. Combining genomic analysis of ISR pathway variants, transcriptomic profiling of stress response activation, proteomic assessment of pathway activity, and metabolomic evaluation of cellular stress states provides holistic patient portraits guiding therapeutic decision-making [49].

The development of companion diagnostics represents a critical need for future ISR therapeutic success, enabling identification of patients with ISR pathway dysregulation who are most likely to benefit from targeted interventions [95]. Real-time monitoring of ISR pathway activity could enable adaptive dosing strategies based on individual response profiles [96].

## 8. Future Perspectives: Beyond Current Limitations

### 8.1. Emerging Technologies and Therapeutic Innovation

CRISPR-based discovery platforms using genome-wide screens in organoids are identifying novel ISR regulators and potential therapeutic targets beyond canonical pathways [97]. These approaches reveal cell-type-specific ISR networks and context-dependent regulatory mechanisms not apparent in traditional systems [98].

Advanced delivery systems including lipid nanoparticles and brain-penetrant prodrugs address blood-brain barrier penetration challenges limiting current compounds [99]. Tissue-specific targeting using engineered delivery vehicles may enable selective ISR modulation in desired cell types while avoiding systemic effects [100].

Network-based interventions targeting multiple stress response nodes simultaneously represent evolution beyond single-pathway approaches [101]. These strategies acknowledge ISR integration with autophagy, mitochondrial quality control, and metabolic networks, potentially overcoming limitations of isolated pathway targeting [102].

### 8.2. Next-Generation Therapeutic Concepts

Moving beyond simple activation/inhibition paradigms, future approaches will emphasize sophisticated modulation strategies [103]. Temporal modulation involves time-specific interventions based on disease stage, preserving early protective functions while targeting later pathological aspects [104]. Combinatorial approaches integrate ISR modulators with standard therapies to enhance efficacy while minimizing resistance [105].

Personalized intervention strategies will use comprehensive biomarker profiles to guide therapeutic decisions [106]. Integration of genetic susceptibility factors, baseline ISR activation status, and disease-specific signatures will enable individualized treatment approaches [92].

The field is evolving toward recognition that ISR represents a dynamic, context-dependent network requiring sophisticated therapeutic strategies [107]. Future success will depend on embracing this complexity rather than seeking simplified solutions [108].

## 9. Critical Limitations and Research Gaps

### 9.1. Fundamental Mechanistic Uncertainties

The molecular switch determining whether ISR activation leads to survival or death remains the field’s most pressing unresolved question [109]. Without understanding this fundamental mechanism, therapeutic targeting remains largely empirical rather than rationally designed [7].

ISR integration with mitophagy, oxidative stress, ER stress, and metabolism represents “the most crucial pathways to unravel” for therapeutic development [110]. The interconnected nature of these systems means that isolated ISR targeting may be insufficient for clinical benefit [111].

The protective-to-pathological transition timeline varies among diseases and remains poorly defined [112]. This knowledge gap complicates therapeutic timing decisions and may explain clinical trial failures [113].

### 9.2. Technical and Translational Barriers

Model system limitations significantly hamper translation [114]. Cell culture findings often fail in vivo translation due to artificial conditions missing critical cell-cell interactions [115]. Short-lived animal models inadequately capture human disease progression, particularly the chronic nature of neurodegenerative processes [116].

Reproducibility crisis: Context dependency makes ISR effects notoriously difficult to reproduce across laboratories [117]. The recent revelation of kinase inhibitor cross-activation calls into question years of published research using these tools at inappropriate concentrations [24].

Drug development challenges: The narrow therapeutic windows observed with ISRIB and other compounds suggest fundamental limitations in current approaches [118]. Achieving sufficient brain penetration while maintaining selectivity remains technically challenging [119].

## 10. Conclusions

The mitochondrial integrated stress response represents one of the most sophisticated cellular signaling networks with profound implications for human health and disease [7]. The 2020 discovery of the OMA1-DELE1-HRI axis fundamentally advanced our understanding of mitonuclear communication [8,9], yet subsequent research has revealed a far more complex picture than originally envisioned.

The field has evolved from simple linear pathway models to recognition of context-dependent networks where cellular metabolic state, temporal dynamics, and disease-specific factors determine ISR outcomes [10]. The concept of molecular switches governing ISR transitions from protection to pathology provides a framework for understanding when stress responses become harmful rather than helpful [110]. Critical thresholds including eIF2α phosphorylation levels, CHOP expression intensity, and response duration determine whether ISR activation promotes cellular adaptation or triggers programmed cell death [111].

The January 2025 clinical trial failures of DNL343 and ABBV-CLS-7262 in ALS mark a watershed moment for the field [11,12]. These setbacks demonstrate that mechanistic understanding alone is insufficient for therapeutic success; patient selection, timing of intervention, disease context, and combination strategies are equally crucial [120]. The emerging understanding that ISR may be predominantly protective in certain disease contexts underscores the importance of disease-specific therapeutic strategies [121].

Future ISR-targeted therapy success depends on precision medicine approaches embracing rather than overlooking this complexity [106]. Biomarker-guided patient selection, combination therapies targeting complementary pathways, and temporal modulation strategies represent the most promising avenues for translating ISR biology into clinical benefit [105]. The development of companion diagnostics and sophisticated patient stratification methods is essential to identify individuals most likely to benefit from ISR-targeted interventions [89].

The dual nature of the mitochondrial ISR—serving as both guardian and executioner depending on cellular context—reflects sophisticated evolutionary pressures shaping this response system [122]. Understanding and therapeutically harnessing this duality represents one of the most promising frontiers in cellular stress biology, with potential applications spanning neurodegeneration, cancer, metabolic diseases, and aging-related pathologies [123]. Continued basic research to understand ISR regulation, innovative therapeutic developments addressing current limitations, and clinical trial designs accounting for the sophisticated biology underlying cellular stress responses will determine whether this fundamental system can be successfully leveraged for human health [124].

## Figures and Tables

**Table 1:** Summary of ISR-Targeted Therapeutics in Clinical Development.

Compound	Mechanism	Target Disease	Phase	Patients (*n*)	Primary Outcome	Status	Reference
DNL343	eIF2B activator	ALS	II/III	325	Failed	Discontinued	[11]
ABBV-CLS-7262	eIF2B activator	ALS	II/III	360	Failed *	Limited continuation	[12]
ISRIB	eIF2B stabilizer	Neurodegeneration	Preclinical	N/A	Mixed	Safety concerns	[48,74,75,76,77,78,79,80]
GSK2606414	PERK inhibitor	Multiple	Preclinical	N/A	Discontinued	Off-target effects	[24,81]

* Exploratory analysis showed subset benefits in muscle strength.

## Abbreviations

The following abbreviations are used in this manuscript:

ALS	Amyotrophic lateral sclerosis
DELE1	DAP3-binding cell death enhancer 1
DELE1L	Full-length DELE1
DELE1S	Short form DELE1
DR5	Death receptor 5
ER	Endoplasmic reticulum
ISR	Integrated stress response
ISRIB	Integrated stress response inhibitor
UPRmt	Mitochondrial unfolded protein response
